# A Manual of Organic Materia Medica

**Published:** 1885-07

**Authors:** 


**A Manual of Organic Materia Medica.** Being a Guide to Materia Medica of the Vegetable and Animal Kingdoms. For the Student, Druggist and Physician. By JOHN M. MAISCH, Phar. D., Prof. of Mat. Med. and Botany in the Philadelphia College of Pharmacy. Second edition. With 242 illustrations. Philadelphia: Lea Brothers & Co. 1885.

This work made its first appearance three years ago, but the demand was so great that the entire edition was exhausted in a year and a half. The author's labors in connection with the last edition of the "National Dispensatory" has prevented an earlier appearance of this new edition.

The present work is a decided improvement upon the previous issue, and will, no doubt, meet with increased favor. To the druggist it is peculiarly valuable.

